# Amyotrophic lateral sclerosis (ALS): new insights 156 years after Charcot's masterful description

**DOI:** 10.1055/s-0046-1827042

**Published:** 2026-08-13

**Authors:** Otto Jesus Hernandez Fustes, Hélio Afonso Ghizoni Teive

**Affiliations:** 1 Universidade Federal do Paraná, Hospital de Clínicas, Departamento de Medicina Interna, Curitiba PR, Brazil.; 2 Universidade Federal do Paraná, Hospital de Clínicas, Departamento de Medicina Interna, Serviço de Neurologia, Curitiba PR, Brazil.

**Keywords:** Amyotrophic Lateral Sclerosis, Jean-Martin Charcot, Neurology

## Abstract

In 1869, Charcot and Alix Joffroy published the first detailed clinical and neuropathological description of amyotrophic lateral sclerosis (ALS), establishing the correlation involving muscle weakness, atrophy, spasticity, and degeneration of the lateral corticospinal tracts. Charcot unified the involvement of upper and lower motor neurons into a single clinical entity. His pioneering description was limited to the motor system, reflecting the scientific constraints of his time. Charcot interpreted ALS primarily as a disorder of the motor system, a conclusion consistent with the clinical and pathological methods available in the late nineteenth century. Neurological investigation at that time relied mainly on detailed clinical observation, anatomical correlation at autopsy, and relatively-simple physiological techniques. These approaches were well suited to identify motor dysfunction but were far less capable of revealing subtle cognitive or behavioral alterations. Currently, ALS is recognized as a multisystem neurodegenerative disorder. Thus, Charcot's historical contribution was crucial for the initial understanding of ALS, while modern perspectives acknowledge its broader clinical complexity beyond the motor system.

Jean-Martin Charcot (1825–1893), a French neurologist considered the father of modern neurology ( Figure 1 ), played a pivotal role in the history of amyotrophic lateral sclerosis (ALS). 1 In 1869, Charcot, in collaboration with Alix Joffroy, 2 published the article “Deux cas d'atrophie musculaire progressive avec lésions de la substance grise et des faisceaux antérolatéraux de la moelle épinière” 3 in the *Archives de Physiologie Normale et Pathologique* . This work provided a detailed description of a previously-unrecognized neurological condition, laying the foundation for the modern understanding of this devastating disease. Their seminal study established the clinicopathological correlation involving muscle weakness and atrophy (lower motor neuron involvement), spasticity (upper motor neuron involvement), and degeneration of the lateral corticospinal tracts.

**Figure 1:**
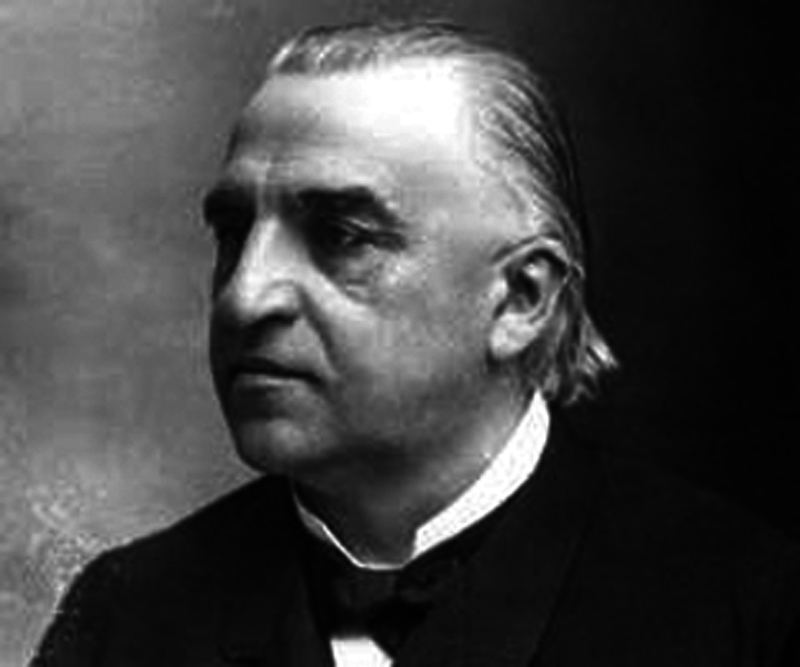
Professor Jean-Martin Charcot (1825–1893). Source: Google Images (Alphabet Inc.).

Charcot and Joffroy identified the progressive degeneration of motor neurons in the motor cortex, brainstem, and spinal cord, correlating the clinical signs (weakness, atrophy, and spasticity) with the underlying anatomical changes. It was the first comprehensive clinical and neuropathological description of what would later be known as *ALS* . Initially, Charcot named the condition *sclérose latérale amyotrophique* , reflecting its pathological hallmarks: Lateral sclerosis—hardening (degeneration) of the lateral corticospinal tracts; and Amyotrophic—muscle wasting due to denervation. We herein present an essential excerpt from the 1869 manuscript: “[...] The descending sclerosis secondary to encephalic lesions is characterized by a rounded focus that exhibits carmine affinity upon staining. Surrounding this focus, the spinal cord tissue remains histologically unremarkable, with a narrow band of preserved white matter. The lesions are bilateral and symmetrical, extending peripherally to the pia mater [...].” 3 The term *sclérose latérale amyotrophique* appears to be established in the twelfth and thirteenth lessons of Charcot's course published by Bourneville (1873), as well as in the subsequent compendia and collected works (1874), thereby solidifying the terminology and the clinical entity as distinct. 4

Charcot's contributions to ALS can be summarized as:

- Precise clinical description: He differentiated ALS from other neurological diseases such as multiple sclerosis and poliomyelitis; 5
- Integration of previously-disparate conditions: Charcot unified *primary amyotrophy* (lower motor neuron) and *primary lateral sclerosis* (upper motor neuron) into a single disease, explaining the mixed signs observed at the bedside; 1
- Clinicopathological correlation: He was a pioneer in demonstrating that the symptoms corresponded to the degeneration of motor neurons; and
- Lasting eponym: In several countries, ALS is referred to as *Charcot's disease* .

In the nineteenth century, Jean-Martin Charcot believed that ALS was a disease confined to the motor system—affecting only the upper motor neurons (in the motor cortex) and lower motor neurons (in the brainstem and spinal cord). This perspective reflected the limited scientific knowledge and diagnostic tools available at the time, as Charcot relied primarily on clinical observation and rudimentary autopsy techniques. He clearly observed motor neuron degeneration and progressive muscle wasting, but had no access to neuroimaging, molecular markers, or electrophysiological methods to detect dysfunction in other parts of the nervous system. The most prominent symptoms were motor-related: muscle weakness, atrophy, spasticity, fasciculations, and paralysis. Because these signs were all obvious and debilitating, non-motor manifestations went largely unnoticed or were attributed to other conditions.

Current evidence indicates that ALS is not exclusively a motor disorder. New evidence has shown that it is a multisystemic disease, including cognitive and behavioral impairments: approximately 50% of the patients present with cognitive alterations, including deficits in executive function, language, and social cognition, while 10–15% develop comorbid frontotemporal dementia (FTD). This multisystem involvement has been defined in new studies that have reported autonomic dysfunction, subtle sensory abnormalities, and affective symptoms such as pseudobulbar affect and emotional lability. The pathogenic mechanisms include several mutations in genes, including *C9orf72* , *TARDBP* , *FUS* , and *SOD1* , which highlights that ALS pathophysiology extends beyond the corticospinal tract, implicating widespread neural networks. 6 7 8 9

Another question concerns the absence of an explicit association between ALS and FTD in Charcot's writings. Although cognitive impairment may have been present in some patients described in nineteenth-century case reports, dementia was generally interpreted as a separate clinical entity rather than as part of the same neurological disorder. In the nosological framework of the time, Charcot's aim was to define a clinicopathological syndrome based on motor symptoms and the degeneration of corticospinal pathways. Consequently, cognitive changes, when present, were not systematically integrated into the description of the disease.

All this new knowledge was not observed in the classic description of Professor Charcot. In the nineteenthth-century medical context, the prevailing paradigm was strongly based on the correlation *anatomical lesion = observable symptom* . Non-motor manifestations were subtle, rarely reported, and difficult to link to central pathology. Furthermore, the ALS–FTD association was not firmly established until the late twentieth century. 10 11

Charcot and his contemporaries also employed early electrophysiological approaches, particularly electrical stimulation of nerves and muscles, which had been introduced into the neurological practice during the nineteenth century. These techniques were primarily designed to assess peripheral nerve and muscle excitability; therefore, they provided limited information about higher cortical functions. As a result, they were unlikely to reveal non-motor aspects of the disease.

Charcot noted that pressure ulcers appeared to be relatively uncommon in patients with ALS when compared with other severely-immobilizing neurological disorders treated at the Salpêtrière. 8 12 13 This observation may reflect the clinical context of large nineteenth-century neurological wards, where patients with spinal-cord injuries, advanced paralysis, or other debilitating conditions frequently developed severe pressure sores. In this setting, ALS may have seemed comparatively less associated with such complications. Charcot attributed this to a presumed *neurotrophic* mechanism, suggesting that the nature of ALS spared the skin from ulceration, unlike other central nervous system disorders. 8 13

However, current clinical evidence does not support Charcot's statement. Modern cohort studies 14 and case reports 15 demonstrate that patients with ALS are at increased risk for pressure ulcers, particularly as immobility progresses. A large nationwide cohort study 14 found that ALS patients have a significantly higher risk of developing pressure sores compared with matched controls (adjusted hazard ratio: 8.82; 95%CI: 4.90–15.9), with an especially high risk in women and younger patients. Case reports 15 also document the occurrence of ischial pressure ulcers in ALS, refuting the notion that these lesions are absent in this population. Reviews 16 of pain and skin complications in ALS further note that, while pressure ulcers may be less common than in other immobilizing diseases, they do occur and require clinical attention.

In summary, Charcot's description of ALS emerged from a rigorous clinicopathological approach that reflected the conceptual and methodological framework of nineteenth-century neurology. His focus on the motor system enabled him to identify the core pathological features of the disease and to distinguish it from other neurological conditions. Subsequent advances in neuropsychology, neuroimaging, and molecular genetics have expanded this original concept, demonstrating that ALS may involve broader neural systems. Rather than contradicting Charcot's observations, these developments illustrate how the progressive refinement of neurological methods has deepened the understanding of the disease he first characterized. ( Table 1 ).

**Table 1:** Evolution of knowledge on amyotrophic lateral sclerosis (ALS)

Period	Discoveries/Advancements	View of ALS
**1869 (Charcot and Joffroy)**	First clinical description: degeneration of upper and lower motor neurons	Exclusively a motor disease
**Early 20th century**	Isolated reports of behavioral changes, but seen as coincidental	ALS still considered solely motor-related
**1940–1960**	Neuropathological studies reveal selective degeneration of motor neurons	Reinforcement of the idea of a pure motor system disease
**1970–1980**	Neuropsychology detects cognitive and behavioral deficits in some patients	First doubts about Charcot's view
**1990s**	Discovery of *SOD1* mutation (1993); protein aggregates in non-motor areas	Evidence that the disease is not restricted to motor functions
**2000s**	Studies show that up to 50% of the patients have cognitive changes; 10–15% develop frontotemporal dementia (FTD)	The ALS–FTD spectrum concept begins to take shape
**2010s**	Discovery of *C9orf72* mutation (2011); confirmation of cognitive and behavioral network involvement	Consolidation of ALS as a multisystem disease
**2020s–present**	Neuroimaging and genetics show motor, cognitive, behavioral, and autonomic involvement	ALS understood as part of a neurodegenerative continuum
